# Prevalence of xerostomia in patients attending Shorish dental speciality in Sulaimani city

**DOI:** 10.4317/jced.51867

**Published:** 2015-02-01

**Authors:** Mustafa Jamel Abdullah

**Affiliations:** 1 B.D.S., M.Sc.Oral Medicine, Oral Medicine Clinic of the school of dentistry, University of Sulaimani, Kurdistan region, Iraq

## Abstract

Objectives: The aim of this study was to investigate the prevalence of xerostomia among dental patients and explore the possible risk factors and symptoms associated with this condition. Patient and Methods: The prevalence of xerostomia and its associations were investigated among patients (n=1132) who were visiting the department of oral medicine at shorish dental speciality in sulaimani city. The age range was between 10-79 years. 512 (45.2%) of participants were males and 620 (54.8%) were females. The data collected were age, sex, systemic diseases, medications and habit of smoking. Logistic regression models to estimate odds ratios and 95% confidence intervals were used to investigate the association of xerostomia with age, systemic diseases and medications and Chi Square test was also used to analyze the data. Results: Prevalence of xerostomia was 16.07%. Prevalence of xerostomia was significantly higher among females (19.51%) than males (11.91%) (P=0.001). The most common diseases with the highest prevalence of xerostomia were psychological disorders (57.14%) followed by diabetes mellitus (53.84%), neurological disorders (40%), thyroid disorders (37.5%) and hypertension (36.48%). The most common medication with the highest prevalence of xerostomia was antihistamine (66.66%) followed by psychotherapeutic medications (60%), pain medications (55.88%), endocrinologic agents (51.21%), antidyslipidic agents (50%) and antihypertensive medication (38.98%). Xerostomia was significantly associated with ageing (OR: 1.02, P=0.000), systemic diseases (OR: 2.80, P=0.000) and medications (OR: 5.17, P=0.000). There was a high prevalence of reported symptoms of xerostomia and these symptoms were more prevalent among females, Prevalence of xerostomia was higher in heavy smoker patients (19.48%) than non smoker patients but not significantly (16.14%) (p= 0.44). Conclusions: There was a high prevalence of xerostomia among dental patients; xerostomia was significantly more prevalent among females and significantly associated with age, systemic diseases and medications; xerostomia adversely affects oral functions; dentist must be familial with sign and symptoms of xerostomia and can have an active role in the management of xerostomia and preventing or treating complications.

** Key words:**Ageing, medications, xerostomia, prevalence.

## Introduction

Xerostomia is a common subjective complaint of dryness in the mouth (1). Self-reported mouth dryness or xerostomia is commonly used in population studies since diagnosing hyposalivation by measurement of saliva flow rates is time-consuming. The amount of saliva needed for a person to experience oral discomfort is on a case by case basis and studies have shown that xerostomia and hyposalivation are not always closely related (2). Saliva is a complex and important body fluid that plays a significant role in the lubrication and protection of oral mucosa, remineralization of teeth, digestion, phonation, taste sensation, buffering action and clearance, and antibacterial activity therefore, this fluid is necessary for the integrity of the oral tissues and is critical for protection and maintaining of oral and dental health (3). Xerostomia has recently been shown to affect the oral health-related quality of life of those affected (4). The presence of xerostomia with a low or altered salivary flow may place patients at a higher risk of dental caries, gingivitis, erosion and ulceration of mucosal tissues, oral candidiasis, dysgeusia and dysphagia (5). A reduction of saliva may lead to complaints of dry mouth, halitosis and oral burning sensation. Other manifestations may include an increasing aversion to dry foods, difficulty with swallowing dry foods, or increased need to sip or drink water when swallowing (6). The major causes of xerostomia among dental patients were the use of medications, and head and neck radiation therapy (7). Other possible causes of dry mouth include uncontrolled diabetes, chronic graft-versus-host disease, sjogren’s syndrome, vasculitis, dehydration, malnutrition, psychogenic conditions and immunodeficiencies (8). The evidence for chemotherapy or normal ageing as causative factors is still controversial (9).

Prevalence of xerostomia was assessed in several other previous investigations in different regions throughout the world (9-11). This study was designed to investigate the prevalence of xerostomia among dental patients and explore the possible risk factors and symptoms associated with this condition.

## Study Design

A cross-sectional survey was carried out among patients (n=1200) who were visiting the department of oral medicine at shorish dental speciality center in Sulaimani city along 4 successive months (January 2014-April 2014) for seeking dental treatment. This research was approved by the Committee of Ethics at research of the University of Sulaimani and Shorish health center. According to declaration of Helsinki, signed consent forms were obtained from all participants before conducting the study (12).

Demographic information such as age and sex were obtained from participants as well as information on the current medical status obtained utilizing in depth interviews; medical conditions were classified into 12 categories and questionnaire was used for each category and the questionnaires were as the following:

1-Do you have any gastrointestinal disorders?

2- Do you have any skeletal disorders?

3-Do you have or have you ever had any cardiovascular disorders such as heart problems, etc?

4- Do you have an endocrine disorders such as diabetes mellitus or thyroid disorders?

5- Do you have any hematologic disorders such as anemia and or a bleeding problem ?

6- Do you have renal disorders such as renal stone, etc?

7- Do you have any allergies?

8- Have you ever had hepatitis, jaundice or liver disease?

9- Do you have or have you ever had any neurological disorders?

10- Do you have or have you ever had psychological disorders such as depression, anxiety and or schizophrenia, etc?

11- Do you have or have you ever had respiratory disorders such as asthma, etc?

12- Do you have any other conditions?

The response options for each questionnaire were ‘yes’ ‘no’ or ‘not sure/maybe’. At the analysis stage, only those who had responded ‘yes’ and suffered from chronic diseases were designated as having medical condition and those who had responded ‘yes’ and suffered from acute condition were not designated as having medical condition. The participants were also asked about medications; “Are you taking any medication?” If yes, then the type of medications were recorded and only medications were currently used for long term treatment were included, medications that were used in the past or it is used symptomatically for short period of time were excluded from the study and they were classified into 14 categories as follows: antihypertensive medication, endocrinologic agents, pain medication, antidyslipidemic agents, antiplatlets medication, gastrointestinal agents, nutritional therapeutics, neurological medications, cardiovascular medication, psychotherapeutic medication, respiratory agents, antihistamine, anticoagulant medication, and others.

In case of the patient was younger than 18 years old, the informed consent was obtained from parents and the parents were asked questionnaire including “Does your child have or have had any of the following conditions?” The twelve questions were explained to the parents and similarly the response options for each question were ‘yes’ ‘no’ or ‘not sure/maybe’. At the analysis stage, only those who had responded ‘yes’ were designated as having medical condition as well as the parents were asked regarding medications including “Is your child taking any medication?” If yes, then the type of medication was recorded.

The participants were asked about habit of smoking too; “do you smoke tobacco products?” If response option was yes, the following question was “how many cigarettes smoked per day? smoker patients were categorized into three groups: light smoker (<10 cigarettes/day), moderate smoker (10-19 cigarettes/day), or heavy smoker (> or =20 cigarettes/day) based on number of cigarettes per day smoked (13). “Former smoker” and “never been smoker” were defined as “nonsmokers” in data analyses. Study members were asked the question “How often does your mouth feel dry?” (Response options ‘Always’ ‘Frequently’ ‘Occasionally’ or ‘Never’). At the analysis stage, those who had responded ‘Always’ or ‘Frequently’ were designated as “xerostomic” (14,15).

Questionnaires were used regarding symptoms of dry mouth which include the following:

1-Do you feel any oral lesion in your mouth?

2-Do you feel burning sensation in your mouth?

3-Do you have a bad breath or halitosis?

4-Do you feel lip cracking?

5-Do you feel difficulties and impairments related to speaking, swallowing and taste sensation?

6-Do you feel dryness in other areas of the body including the lip, throat, eye, nose, and the skin?

7-Do you feel you need to wake up to drink water?

8- Do you feel thirsty?

9- Do you feel difficulty eating dry food?

The response options for each questionnaire were ‘yes’ ‘no’ or ‘do not know’. At the analysis stage, only those who had responded ‘yes’ were recorded.

In this study patients who were mentally retarded or were unable to answer questionnaire due to other conditions such as deafness etc were also excluded from the study.

Statistical analysis was performed using SPSS program version 16. Associations between categorical variables were tested using chi-square test; logistic regression test was used to determine association between xerostomia and age, systemic diseases and medications. Statistical significance was set at *P* < 0.05.

## Results

The sample comprised 1200 patients but 68 patients rejected to participate therefore the final number was 1132 participants aged from 10-79 years. 512(45.22) of participants were male and 620 (54.77) were female. Female to male ratio was 1,2:1 with mean age±SD=32.32±13.18. They were categorized into four age groups: less than twenty years, twenty to thirty nine years, forty to fifty nine years and equal and more than sixty years. More than half of participants (57.68) were in age group 20-39 years and the smallest prevalence of participants were in age group equal and more than sixty years (4.24%) followed by less than twenty years (15.63%). There were no statistically significant sex differences between each consecutive age groups (*p*>0.05).

Prevalence of xerostomia was 16.07%. The prevalence of xerostomia was more or less linearly increased with increasing age with highest prevalence in the age group equal and more than sixty years (33.33) were it showed significantly higher prevalence of xerostomia than age group twenty to thirty nine years (14.70%). Logistic regression test showed statistically significant association between age and developing xerostomia (OR: 1.02, *P*=0.000) and also when male and females were considered separately; among females logistic regression test showed statistically significant association between age and developing xerostomia (OR: 1.02, *P*=0.000); on the other hand among males logistic regression test showed no association between xerostomia and age (OR: 1.00, *P*=0.34). Over all prevalence of xerostomia was significantly higher among females (19.51%) than males (11.91%) (*P*=0.001) ( Table 1).

**Table 1:**
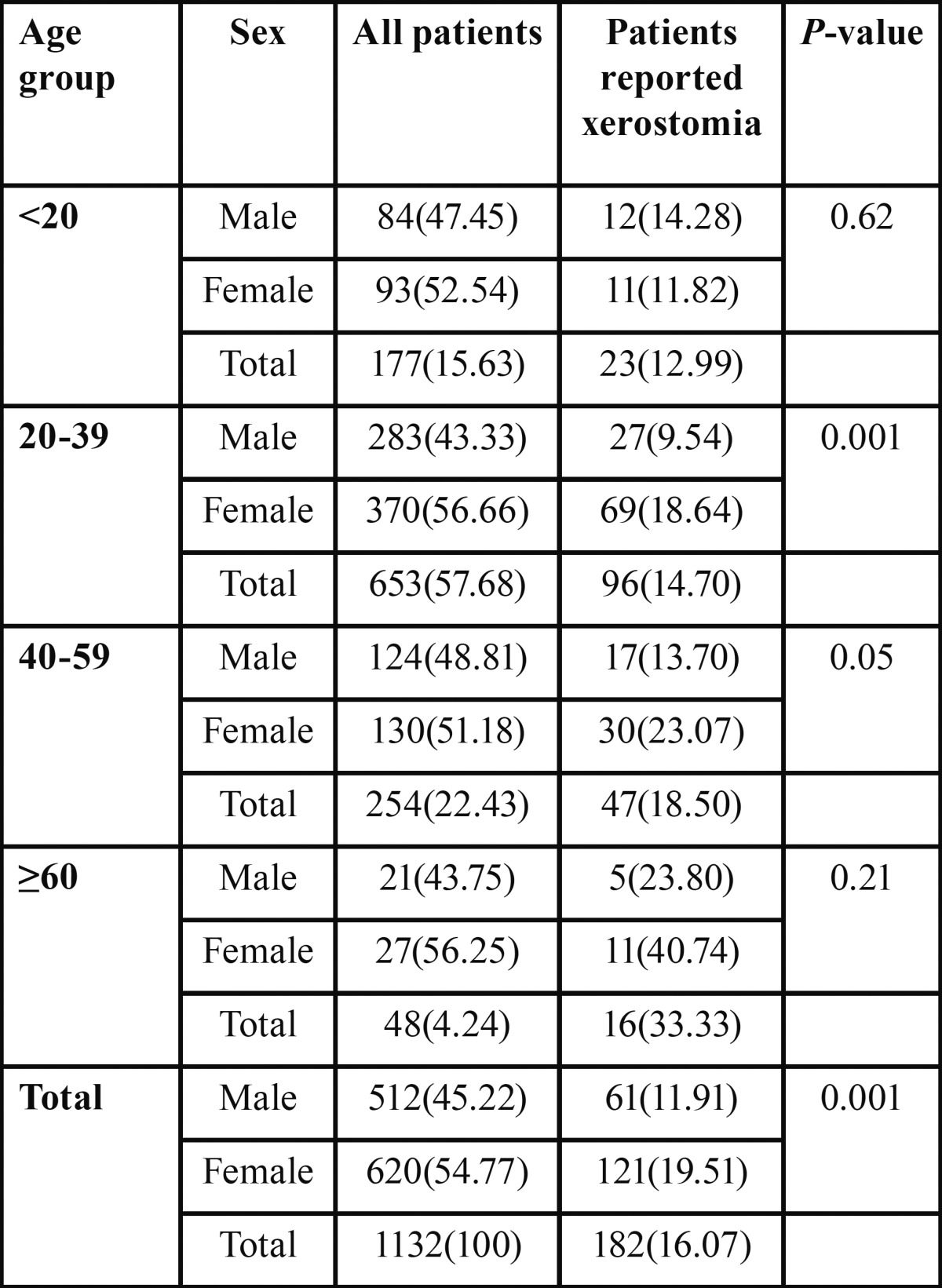
Prevalence of xerostomia according to age and sex.

The most common diseases with the highest prevalence of xerostomia were psychological disorders (57.14%) followed by diabetes mellitus (53.84%), neurological disorders (40%), thyroid disorders (37.5%) and hypertension (36.48%). Prevalence of xerostomia was higher among patients with diseases than patients without diseases in all disease categories except liver disorders and respiratory disorders; the difference were statistically significant (*P*<0.05) except hematological disorders, renal disorders, and allergy (*P*>0.05). Overall patients with diseases were three times more likely to report xerostomia (OR: 2.8, 95% CI 2-3.8) than patients without diseases. Logistic regression test showed statistically significant association between disease and xerostomia (OR: 2.80, *P*=0.000) ( Table 2).

**Table 2:**
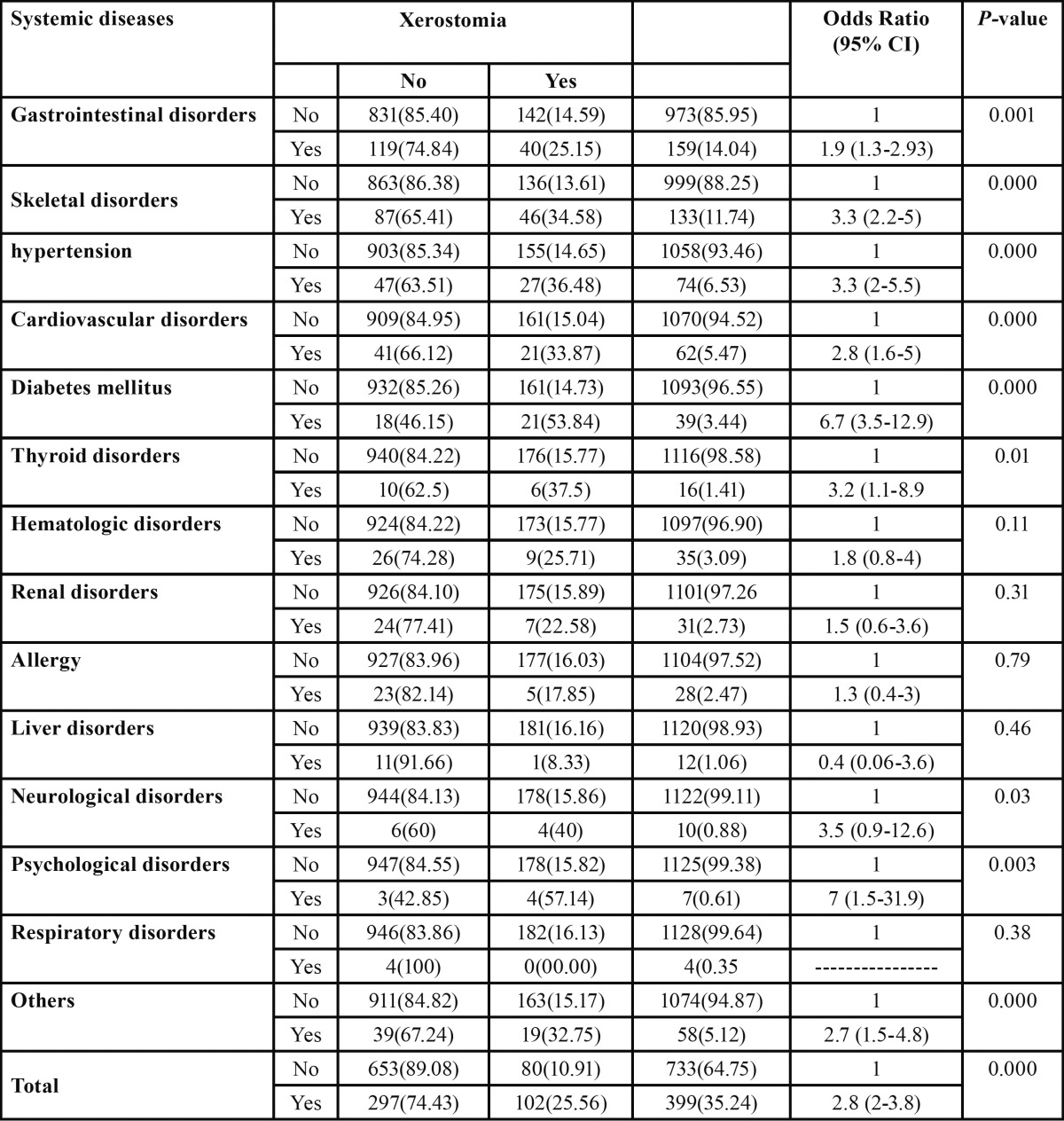
Comparison of prevalence of xerostomia between patients with medical condition and patients without medical condition.

The most common medication with the highest prevalence of xerostomia was antihistamine (66.66%) followed by psychotherapeutic medications (60%), pain medications (55.88%), endocrinologic agents (51.21%), antidyslipidic agents (50%), and antihypertensive medication (38.98%).

Prevalence of xerostomia was higher among patients who were using medications than patients who were not using medications in all medication categories but only antihypertensive medication, endocrinologic agents, pain medication, antidyslipidemic agents, psychotherapeutic medication, and antihistamine were significantly showed higher prevalence of xerostomia than patients who were not using these medications.

Overall patients who were using medications were five times more likely to report xerostomia ( OR: 5.1, 95% CI 3.5-7.4) than patients who were not using medications. Logistic regression test showed statistically significant association between use of medications and xerostomia (OR: 5.17, *P*=0.000) ( Table 3).

**Table 3:**
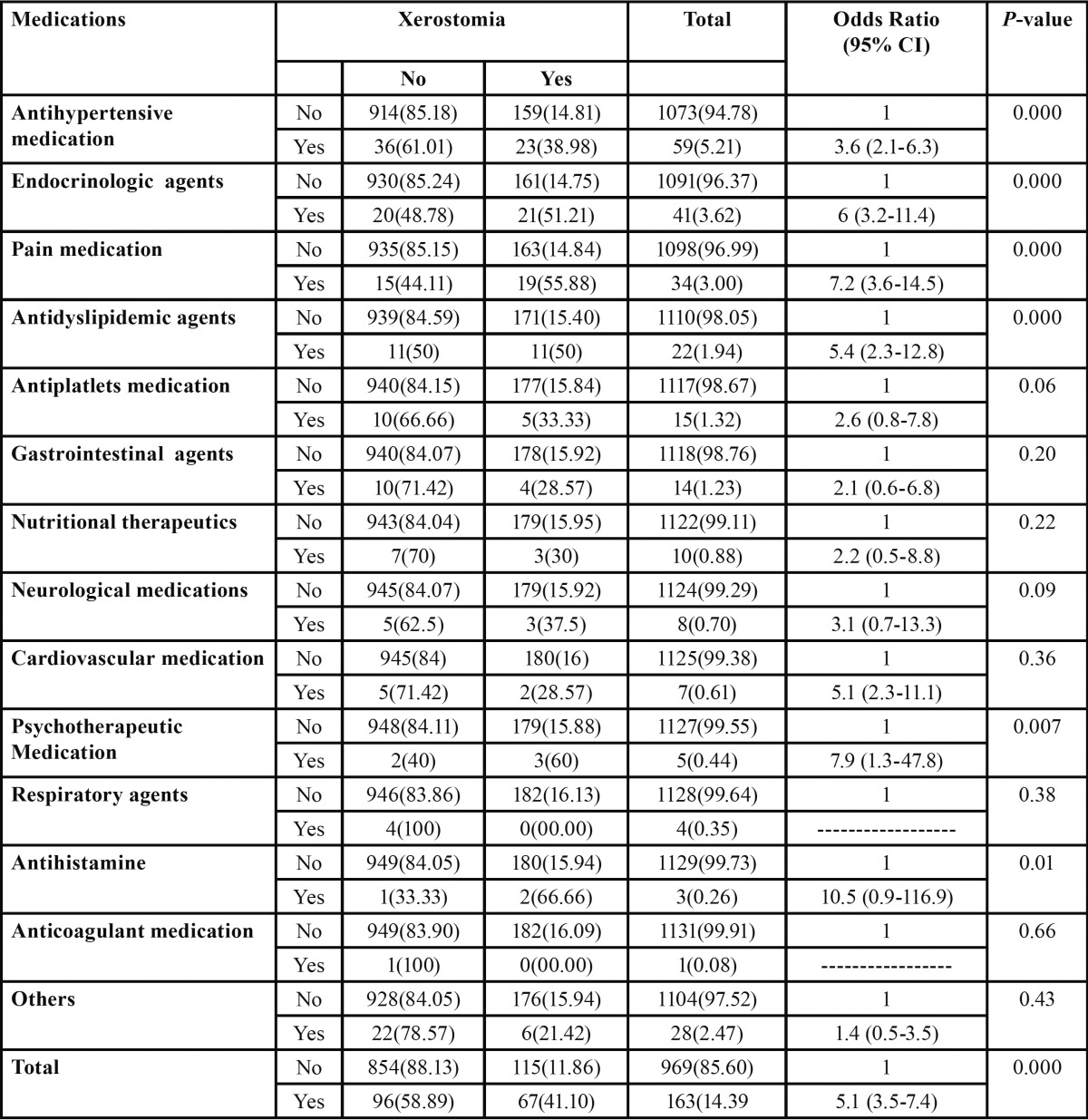
Comparison of prevalence of xerostomia between patients on medications and patients not on medications.

**Figure 1:**
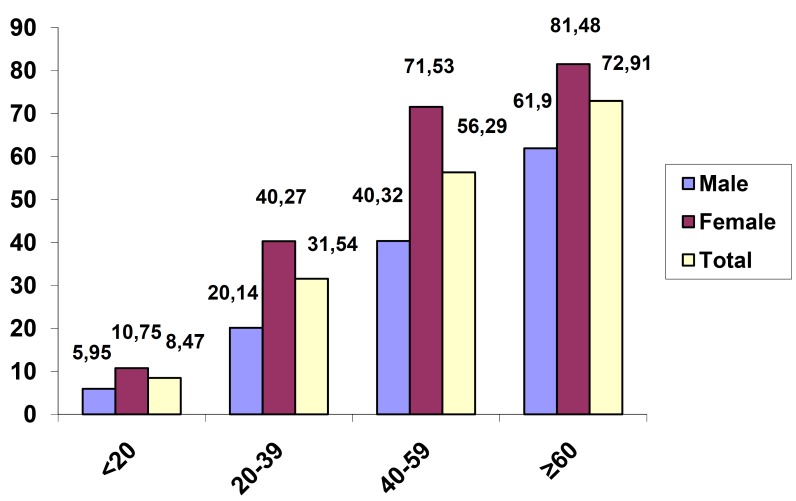
Prevalence of medical conditions according to age and sex.

**Figure 2:**
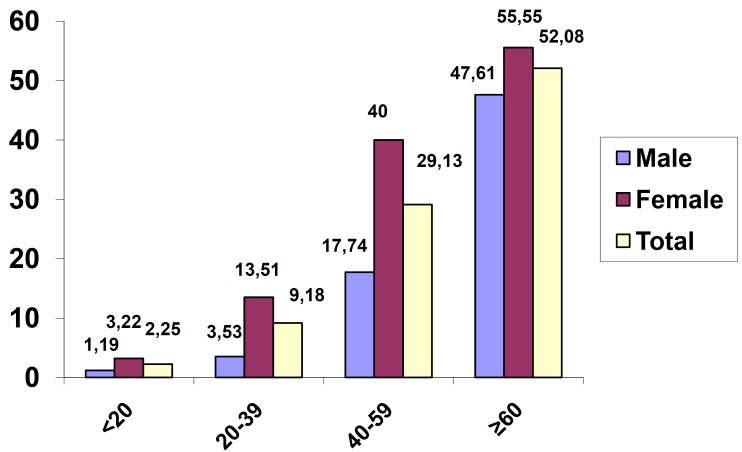
Prevalence of medication use according to age and sex.

Prevalence of medical condition was increased significantly with increasing age (*P*<0.001), (*P*=0.03 for age group equal and more than sixty years versus age group forty to fifty nine years). Prevalence of medication use was increased significantly with increasing age (*P*<0.001) (Figs. 1,2). Prevalence of medical condition and medication use were significantly higher among females than males (*P*<0.001) (Figs. 1,2).

There was a high prevalence of reported symptoms among xerostomic patients. Prevalence of reported symptoms were higher among females than males except self reported oral lesion, self perceived taste disturbance and dry lip but only self reported burning mouth was significantly higher among females (18.18%) than males (4.91%) (*p*=0.01) ( Table 4).

**Table 4:**
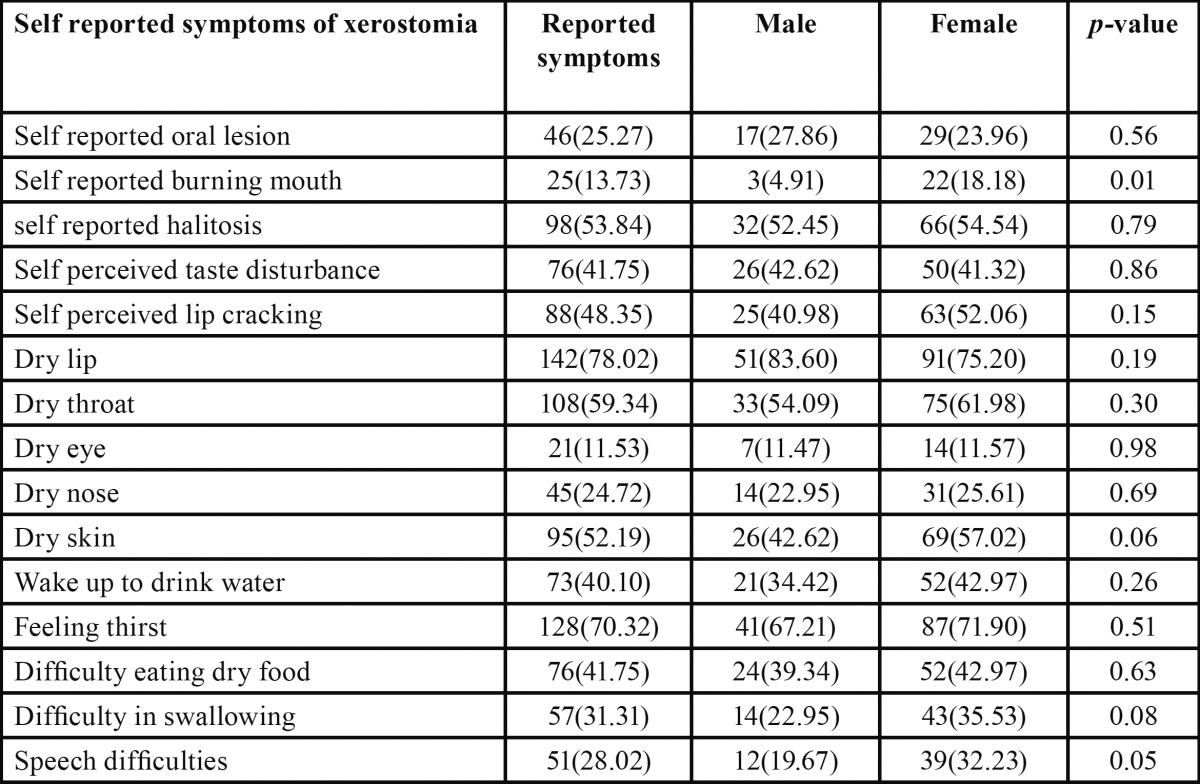
Prevalence of reported symptoms among 182 patients reported xerostomia according to sex.

**Figure 3:**
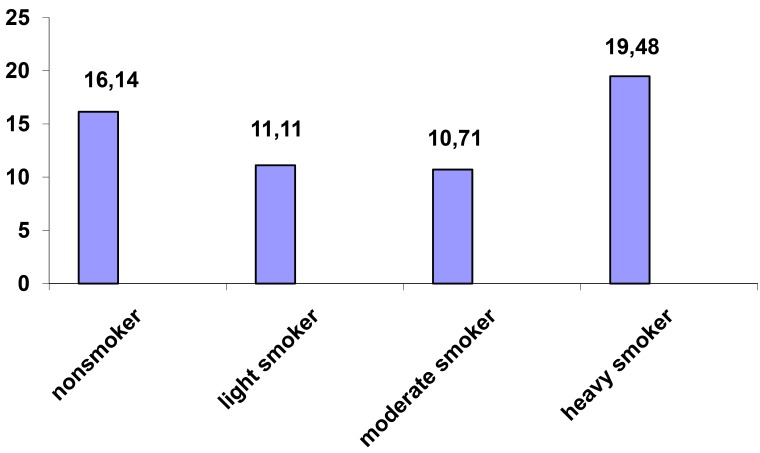
Prevalence of xerostomia according to habit of smoking.

Prevalence of xerostomia was higher in heavy smoker patients (19.48%) than non smoker patients but not significantly (16.14%) (*p*= 0.44) (Fig. 3).

## Discussion

Most studies on xerostomia have been carried out on community-dwelling or institutionalized adults, very few studies and data are available for people under age 50 years especially in very young populations. The present study was a cross-sectional evaluation of prevalence of xerostomia in a wide age- range from 10 to 79 years of age.

This study investigated the prevalence of and risk factors of xerostomia among a large number of dental patients. In a systematic review in Scandinavia, the prevalence of xerostomia in the 13 articles ranged from 0.9% to 64.8% (16). In all articles, the xerostomia level was determined by asking subject specific questions about dry mouth. In this study prevalence of xerostomia was 16.07%. This result is comparable to other studies done by Hahnel *et al.* (10) (16%), Villa and Abati (9) (19.6%) and it was less than that reported in another study done by So *et al.* (11) (70.1%). The variations in the prevalence of xerostomia in different studies may be because of many factors such as age, sex, sample size and methodology used to carry out the study including the number, the content of the questions, and the guidelines used for the diagnosis of xerostomia.

In this study, as expected the prevalence of xerostomia was more or less linearly increased with increasing age with highest prevalence in the age group equal and more than sixty years were it showed significantly higher prevalence of xerostomia than age group twenty to thirty nine years. Logistic regression test showed statistically significant association between age and developing xerostomia. Similar result were reported in several other previous investigations (9,17,18).

In this study, the enlarged prevalence of xerostomia with increasing age may be because of significant raise in prevalence of medical condition and use of medication with increasing age. Other possible factors are age-related changes in the composition of saliva (19) and geriatric malnutrition (20).

Prevalence of xerostomia was significantly higher among females than males. Among females logistic regression test showed statistically significant association between age and developing xerostomia but not among males. Similarly in the majority of the other studies female had experienced more symptoms of xerostomia than male (17,18) however, in a study by Murray *et al.* (15) no appreciable sex difference was observed. This might be related to the menopausal age of the women (21) in this regard, it has been suggested that both quantity and quality of saliva will be influenced by the menopause in women, thus being potentially important for xerostomia. Some studies on healthy women have reported higher salivary secretion before menopause than after (22) whilst others did not find any difference (23). The different composition of saliva in peri- and post-menopausal women has been suggested to be depending on the amount of oestrogen (24) and the fact that women tend to report higher pain intensity in general and are more expressive about their general illness condition (25). Additionally, dry mouth may be affected by psychological status, such as anxiety and depression (26), as the prevalence of psychological symptoms or diseases were reported to be higher in women than in men (27). In this study, the higher prevalence of xerostomia among females than males may be because of significantly higher prevalence of medical condition and use of medication among females than males.

There were several reports claiming that the main causes for xerostomia and hyposalivation were systematic diseases and medication (18,28) which also was supported in this study.

In this study, logistic regression test showed xerostomia was significantly associated with systemic diseases; patients with diseases were three times more likely to report xerostomia than patients without diseases and the most common diseases with the highest prevalence of xerostomia were psychological disorders followed by diabetes mellitus, neurological disorders, thyroid disorders and hypertension. Similarly in another study done by Villa and Abati (9) individuals with nervous or mental disorders reported highest prevalence of xerostomia.

Several drug groups such as antidepressants, anticholinergetics and antihistamines have been associated with xerostomia in multiple studies (29).

In this study, logistic regression test showed xerostomia was significantly associated with medications; patients who were using medications were five times more likely to report xerostomia than patients who were not using medications and the most common medication with the highest prevalence of xerostomia was antihistamine followed by psychotherapeutic medications, pain medications, endocrinologic agents, antidyslipidic agents, and antihypertensive medication.

There was a high prevalence of reported symptoms among xerostomic patients; with some exceptions prevalence of reported symptoms were higher among females than males. These symptoms could be used for diagnostic criteria of oral dehydration and salivary gland hypo-function (30).

The findings of this study suggest that patients suffering from xerostomia may experience dry lips, throat, eyes, skin and nose. These symptoms could alert the dentist to a more serious problem, such as Sjogren’s syndrome. Dentists may be the first health care providers to encounter the early signs of Sjogren’s syndrome therefore, they should be familiar with the manifestations of the disease and be prepared to take an active role in the diagnosis, management and treatment of the oral complications and eventually consult a rheumatologist if the symptoms persist (9).

Prevalence of xerostomia was higher in heavy smoker patients than non smoker patients but not significantly. Similarly in another study done by Villa and Abati (9) no significant association was observed between smoking and xerostomia; on the other hand majority of other studies showed significant association between smoking and xerostomia (17,18).

In summary, the result of this study showed high prevalence of xerostomia among dental patients. Xerostomia was significantly more prevalent among females and significantly associated with age, systemic diseases and medications. Xerostomia can have an adverse effect on oral functions such as taste sensation, swallowing, speech, etc, and it may negatively affect general well being and quality of life, and oral health. There was a high prevalence of reported symptoms of xerostomia among dental patients therefore these reported symptoms could be used for diagnosis of xerostomia; with some exceptions symptoms were more prevalent among females and were more prevalent among heavy smokers than non smokers although without statistical significance; dentist must take careful history and clinical examinations for management of xerostomia using a tailored approach to find patients main risk factors and preventing or managing complications.
